# Evaluation of Adverse Events Associated with the Sulfamethoxazole/Trimethoprim Combination Drug

**DOI:** 10.3390/jcm14144819

**Published:** 2025-07-08

**Authors:** Takaya Sagawa, Tomoaki Ishida, Kohei Jobu, Shumpei Morisawa, Keita Akagaki, Takahiro Kato, Takumi Maruyama, Yusuke Yagi, Tomomi Kihara, Sanae Suzuki, Mio Endo, Nobuaki Matsunaga, Yukihiro Hamada

**Affiliations:** 1 Department of Pharmacy, Kochi Medical School Hospital, 185-1 Kohasu, Oko-cho, Nankoku-shi 783-8505, Kochi, Japan; jm-tky-s@kochi-u.ac.jp (T.S.); jm-kouheij@kochi-u.ac.jp (K.J.); jm-morisawa.s@kochi-u.ac.jp (S.M.); jm-keita_akagaki@kochi-u.ac.jp (K.A.); jm-takkato@kochi-u.ac.jp (T.K.); jm-maruyama.takumi@kochi-u.ac.jp (T.M.); jm-yyagi@kochi-u.ac.jp (Y.Y.); 2 Graduate School of Integrated Arts and Sciences, Kochi University, Kohasu, Oko-cho, Nankoku-shi 783-8505, Kochi, Japan; 3 Department of Clinical Pharmacy, Graduate School of Pharmaceutical Sciences, Nagoya City University, 3-1 Tanabe-dori, Mizuho-ku, Nagoya 467-8603, Japan; t-ishida@phar.nagoya-cu.ac.jp; 4 Department of Infection Prevention and Control, Kochi Medical School Hospital; 185-1 Kohasu, Oko-cho, Nankoku-shi 783-8505, Kochi, Japan; 5 Division of Public Health, Department of Planning and Coordination, Bureau of Health Security and Management, Japan Institute for Health Security, 1-23-1 Toyama, Shinjuku, Tokyo 162-8640, Japan; kihara-t@niid.go.jp; 6 Department of Public Health Medicine, Institute of Medicine, and Health Services Research and Development Center, University of Tsukuba, 1-1-1 Tennodai, Tsukuba 305-8575, Ibaraki, Japan; 7 AMR Clinical Reference Center, National Center for Global Health and Medicine Japan Institute for Health Security, 1-21-1 Toyama, Shinjuku, Tokyo 162-8655, Japan; suzuki.sana@jihs.go.jp (S.S.); endo.mi@jihs.go.jp (M.E.); matsunaga.no@jihs.go.jp (N.M.)

**Keywords:** Japanese adverse drug event report, spontaneous reporting system, sulfamethoxazole/trimethoprim, confidence intervals, Weibull

## Abstract

**Background/Objectives**: The combination drug sulfamethoxazole/trimethoprim (ST) is a broad-spectrum antibiotic used against various infections; however, it is associated with several serious adverse events. The ST package inserts contain warnings about these adverse events. However, warnings vary internationally, and specific measures to address ST-related adverse events are unclear. Therefore, we aimed to comprehensively evaluate ST-related adverse events using the Japanese Adverse Drug Event Report (JADER) database and analyze the onset time for each event. **Methods**: Adverse events due to ST were analyzed using the JADER database between April 2004 and June 2023. The reported odds ratio and 95% confidence interval (95% confidence interval [CI]) were calculated, with a signal detected if the 95% CI lower limit exceeded 1. The Weibull distribution was used to characterize the onset time of adverse events with detected signals. **Results**: The total number of cases in the JADER database during the study period was 862,952, and the number of adverse events involving ST as a suspected drug was 4203. Adverse events associated with ST include hyperkalemia, syndrome of inappropriate antidiuretic hormone secretion, hematopoietic cytopenia, acute renal failure, hypoglycemia, disseminated intravascular coagulation syndrome, hepatic disorder, and the Stevens–Johnson syndrome/toxic epidermal necrolysis. **Conclusions**: Weibull analysis indicated an early failure-type onset time for all adverse events, suggesting the need for intensive adverse event monitoring of ST, especially in the first month of use. These findings may support revising drug package inserts in Japan to better reflect the identified risks.

## 1. Introduction

The sulfamethoxazole/trimethoprim (ST) combination drug comprises two antibiotics: sulfamethoxazole, a sulfonamide that inhibits dihydropteroate synthase, and trimethoprim, an aminopyrimidine that inhibits dihydrofolate reductase. Together, they disrupt tetrahydrofolate biosynthesis, inhibit thymidine synthesis, and exert bacteriostatic effects against bacteria and fungi [1,2]. Trimethoprim has been associated with dose-dependent bone marrow suppression through the inhibition of folate synthesis [3,4,5]. When combined with sulfamethoxazole, the two drugs exert a synergistic antimicrobial effect by sequentially inhibiting two key enzymes in the bacterial folate synthesis pathway: dihydropteroate synthase and dihydrofolate reductase, respectively. This dual blockade more effectively suppresses tetrahydrofolate synthesis, thereby enhancing bacteriostatic activity across a broad range of pathogens. The synergistic mechanism not only broadens the antimicrobial spectrum but also reduces the likelihood of resistance development and permits lower individual drug dosages compared with those in monotherapy [6,7]. ST exhibits a broad spectrum of antimicrobial activity against Gram-positive bacteria, Gram-negative bacteria, and certain fungi. According to guidelines from the Infectious Diseases Society of America (IDSA), ST is recommended as a treatment option for mild-to-moderate skin and soft tissue infections, including those caused by community-acquired methicillin-resistant *Staphylococcus aureus* [8]. Additionally, the Centers for Disease Control and Prevention, as well as the IDSA, recommend ST as a first-line agent for the treatment and long-term prophylaxis of *Pneumocystis pneumonia*, especially in immunocompromised individuals. These include individuals with HIV/AIDS, patients undergoing solid organ or hematopoietic stem cell transplantation, those receiving cytotoxic chemotherapy, and those on prolonged high-dose corticosteroids [9,10,11]. Furthermore, ST is widely used in the treatment of urinary tract infections and *Nocardia* infections, and as prophylaxis in patients with chronic granulomatous disease. While ST remains a central antimicrobial agent, recent studies have raised concerns regarding the emergence of *Staphylococcus aureus*-resistant strains, including small colony mutants [12,13], particularly in monotherapy, and caution should be exercised when using it long-term. However, ST use is associated with adverse events, which can make treatment continuation difficult. Notably, hematopoietic cytopenia and renal failure are frequently reported adverse events [3,14]. In addition, severe adverse events with a low incidence, such as Stevens–Johnson syndrome/toxic epidermal necrolysis (SJS/TEN), syndrome of inappropriate antidiuretic hormone secretion (SIADH), and severe hypoglycemia, have also been reported. Despite these risks, studies investigating ST-associated adverse events are limited, and the causal relationship remains unclear [15,16,17]. Against this background, side effect warnings have been included in the package inserts of various countries. In Japan, warnings for anaphylactic reactions and hematopoietic cytopenia are listed, while SJS/TEN is identified as a serious adverse effect in the United States and the United Kingdom [18,19,20]. These adverse events are severe and potentially fatal, necessitating measures for their prevention and early detection. To promptly identify these effects and take appropriate action, such as discontinuing or switching medications or providing symptomatic treatment, it is crucial to understand when these events are most likely to occur after treatment begins. However, to our knowledge, no study has statistically evaluated the onset timing of ST-related adverse events using a large database. Existing reports on onset timing are limited to isolated case reports, which do not permit generalizable conclusions or estimates of typical patterns of onset.

We hypothesized that ST-related adverse events would follow an early-onset pattern of clinical significance. To test this hypothesis, we conducted a Weibull analysis using the Japanese Adverse Drug Event Report (JADER), a national database of adverse drug reactions, to validate the onset pattern. Therefore, the aim of this study was to evaluate the detection of ST-related adverse events using the JADER database and determine their timing, thereby clarifying the appropriate monitoring period for each adverse event. Additionally, we aimed to generate information not currently included in the package inserts regarding adverse events associated with ST use in Japan and to provide information for revision of the package inserts.

## 2. Materials and Methods

### 2.1. Data Extraction

The JADER database, which is maintained by the Pharmaceuticals and Medical Devices Agency (PMDA), is a collection of voluntary adverse drug reaction reports. Data recorded in the JADER database between April 2004 and June 2023 were obtained from the PMDA website (http://www.pmda.go.jp/; accessed on 10 January 2024). The JADER dataset includes four tables containing the following: (1) patient information (sex, age, and weight); (2) drug information; (3) adverse events and outcomes; and (4) medical history and underlying conditions. These tables were merged using the Fund E-Z Backup Archive v11. 06. 01(Fund E-Z Development Corporation, White Plains, NY, USA) [21].

Overall, 862,952 cases in the JADER dataset were analyzed, and the reported odds ratios (RORs) and 95% confidence intervals (CIs) for ST-related adverse events were calculated using data from these cases. To analyze drug-induced adverse events, cases in which ST was administered orally or intravenously were selected from each dataset. Among these, we selected 4203 cases in which ST was identified as a suspected drug.

### 2.2. Analysis of the ROR

In JADER, each drug entry includes a classification for “drug involvement”, categorized as “suspect drug,” “concomitant drug,” and “drug interaction.” In this analysis, only cases classified as “suspect drug” were considered indicative of suspected adverse events, while cases involving “concomitant drug” and “drug interaction” were analyzed separately.

The International Council for Harmonisation of Technical Requirements for Pharmaceuticals for Human Use (ICH) and the Medical Dictionary for Regulatory Activities (MedDRA) v24.0 were used to extract adverse events and underlying diseases recorded in the JADER database. We extracted cases in which ST was the suspect drug and counted all adverse events at the MedDRA Preferred Term code (PT) level. The top 40 events were ranked by the number of reports, and included in the analysis. To avoid duplication of similar events, we merged the relevant PTs with reference to the Standardised MedDRA Queries (SMQ) and calculated the ROR for the merged events (Table A1).

RORs and 95% CIs were calculated to analyze ST-associated adverse events. RORs were determined by classifying cases into four categories: (a) individuals administered ST who developed the target adverse events; (b) individuals administered ST who developed other adverse events; (c) individuals administered other drugs who developed the target adverse events; and (d) individuals administered other drugs who developed other adverse events. RORs and 95% CIs were calculated using the following formulae:

ROR=(a/c) / (b/d),

95% CI=exp (log (ROR) ± 1.96 √((1/a)+(1/b)+(1/c)+(1/d))),

RORs were expressed as point estimates with 95% CIs, and statistical analysis was performed using Fisher’s exact test. A signal was detected if the lower limit of the 95% CI of the crude ROR exceeded 1.

The adjusted ROR was calculated based on previous reports of adverse events with the ST combination drug as the suspect drug. Overall, 762,191 cases were included in the analysis, excluding those with missing age and gender reporting. Gender (female), reporting year, and stratified age groups (0–19, 20–29, 30–39, 40–49, 50–59, 60–69, 60–70, and 80+) were coded to construct the logistic model.

$$\mathrm{Log}\left(odds\right)=\beta_{0}+\beta_{1}Y+\beta_{2}S+\beta_{3}A+\beta_{4}D$$

$$\left(Y=reporting\;year,|S=sex,|A=stratified\;age\;group,|D=drug\right)$$

All analyses were conducted using EZR (version 1.51, Saitama Medical Center, Jichi Medical University, Saitama, Japan). Statistical significance was set at *p* < 0.05. To ensure transparency in annual variations, the total number of reports per year and the percentage of reports related to ST were presented (Table A2).

### 2.3. Analysis of Onset Time of Adverse Events

We selected 2359 cases (56% of 4203 total cases) in which ST was the suspected drug. Records of the occurrence of complete adverse events and prescription start dates were used for time-to-onset analysis. The onset of adverse events was calculated from the first prescription to the occurrence of adverse events. The median duration, interquartile range (IQR), and Weibull shape parameters were used to analyze the onset data [22,23]. The Kaplan–Meier method was used to illustrate the cumulative incidence of adverse drug events associated with ST [24]. The Weibull shape parameter alpha (α) is the scale of the distribution, with a larger scale indicating a stretched distribution, whereas smaller-scale values shrink the data distribution. The Weibull shape parameter beta (β) defines the shape of the distribution function. The shape parameter β value categorized failure into three groups: if β = 1, the hazard was estimated to be constant over time (random failure type); if β > 1 (with 95% CI greater than 1), the hazard was considered to increase with time (wear-out failure type); and if β and the 95% CI were lower than 1, the hazard was considered to decrease with time (early failure type) [25]. These analyses were conducted using JMP 14.0 (SAS Institute, Mumbai, India).

## 3. Results

### 3.1. RORs and Number of Cases of Each Adverse Event Associated with ST

The ROR (95% CI) for each adverse event associated with ST in the JADER database was calculated. Signals for adverse events with ST as the suspected drug were detected for hyperkalemia (ROR: 11.3; 95% CI: 9.85–13.0), SIADH (ROR: 4.67; 95% CI: 3.90–5.55), hematopoietic cytopenia (ROR: 3.53; 95% CI: 3.31–3.79), hypersensitivity (ROR: 2.78; 95% CI: 2.59–2.97), acute renal failure (ROR: 2.39; 95% CI: 2.12–2.68), hypoglycemia (ROR: 2.33; 95% CI: 1.89–2.84), and hepatic disorder (ROR: 2.07; 95% CI: 1.90–2.26) (Table 1). In contrast, signals for SIADH (ROR: 1.37; 95% CI: 1.18–1.58), hematopoietic cytopenia (ROR: 3.79; 95% CI: 3.67–3.91), acute renal failure (ROR: 1.13; 95% CI: 1.04–1.21), and hepatic disorder (ROR: 1.07; 95% CI: 1.01–1.12) were also detected as concomitant or interaction drug signals (Table 1).

Hematopoietic cytopenia was analyzed by subdividing the reduction in each type of hematopoietic cell. Signals for ST as the suspected drug were detected for pancytopenia (ROR: 4.22; 95% CI: 3.75–4.75), erythropenia (ROR: 4.06; 95% CI: 2.93–5.50), leukopenia (ROR: 2.80; 95% CI: 2.58–3.04), and thrombocytopenia (ROR: 2.56; 95% CI: 2.28–2.86), with similar signals observed for concomitant or interacting drugs (Table 1).

Hypersensitivity was analyzed by subdividing the data according to the allergy type. A signal for ST as the suspected drug was detected for SJS/TEN (ROR: 7.19; 95% CI: 6.41–8.04); however, no signal was identified for anaphylactic reactions. No signals were observed for ST as a concomitant or interacting drug in either anaphylactic reactions or SJS/TEN (Table 1).

The adjusted ROR was calculated for each adverse event with ST as the suspect drug using a logistic regression model adjusted for age, gender, and year of report as confounders. The results showed elevated adjusted RORs for several events, including hyperkalemia (adjusted ROR: 9.45; 95%CI: 8.10–1.10), SIADH (adjusted ROR: 4.75; 95%CI: 3.98–5.65), hematopoietic cytopenia (adjusted ROR: 3.56; 95%CI: 3.32–3.81), cytopenia affecting more (adjusted ROR: 4.35; 95%CI: 3.87–4.90), erythropenia (adjusted ROR: 3.65; 95%CI: 2.65–5.02), leukopenia (adjusted ROR: 2.79; 95%CI: 2.57–3.03), thrombocytopenia (adjusted ROR: 2.45; 95%CI: 2.19–2.76), hypersensitivity (adjusted ROR: 2.66; 95%CI: 2.48–2.86), SJS/ TEN (adjusted ROR: 6.74; 95%CI: 6.01–7.56), acute renal failure (adjusted ROR: 2.21; 95%CI: 1.95–2.50), hypoglycemia (adjusted ROR: 2.41; 95%CI: 1.96–2.96), and hepatic disorder (adjusted ROR: 1.98; 95%CI: 1.81–2.17). The same adverse events identified in the crude ROR were also signaled in this model (Table 2).

### 3.2. Time-to-Onset Analysis by the Weibull Distribution of Adverse Events

Records with complete dates of ST adverse events and prescription start dates (2359 cases, 56% of 4203 total cases) were used for the time-to-onset analysis.

**Figure 1:**
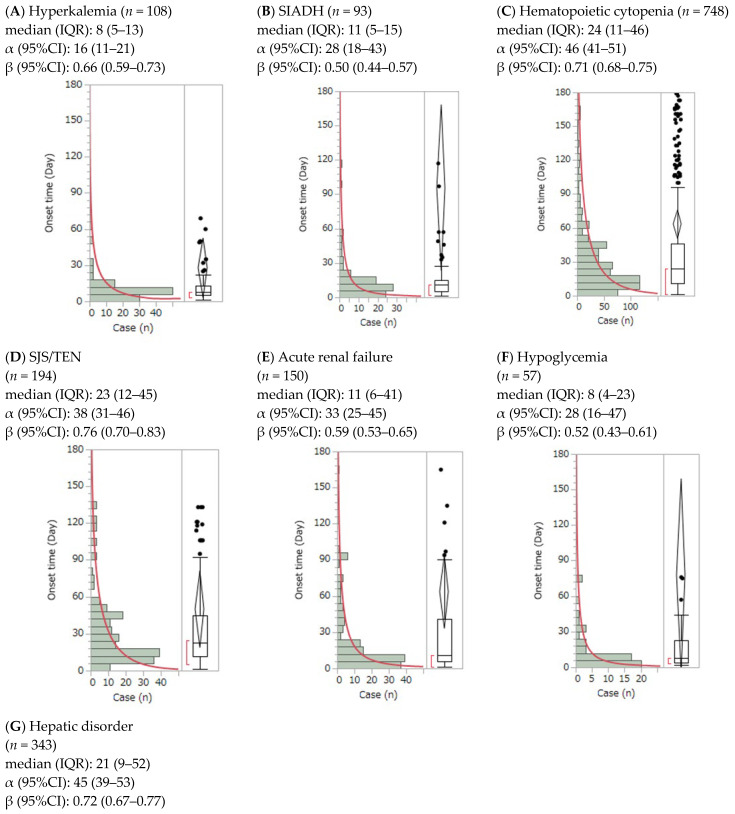

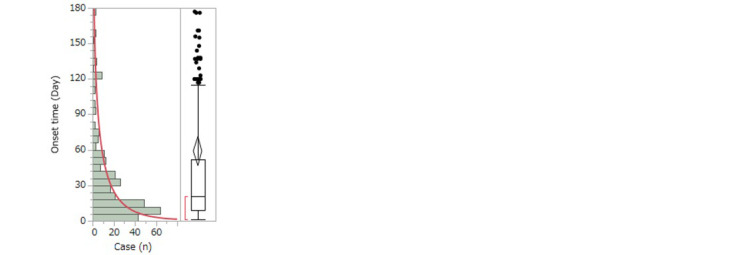
Time-to-onset analysis of adverse events by sulfamethoxazole/trimethoprim administration. Histograms for (**A**) hyperkalemia, (**B**) syndrome of inappropriate secretion of antidiuretic hormone, (**C**) hematopoietic cytopenia, (**D**) Stevens–Johnson syndrome/toxic epidermal necrolysis, (**E**) acute renal failure, (**F**) hypoglycemia, and (**G**) hepatic disorders are shown. Abbreviations: 95% CI, 95% confidence interval; IQR, interquartile range; SIADH, syndrome of inappropriate antidiuretic hormone secretion; SJS/TEN, Stevens–Johnson syndrome/toxic epidermal necrolysis.

**Figure 2:**
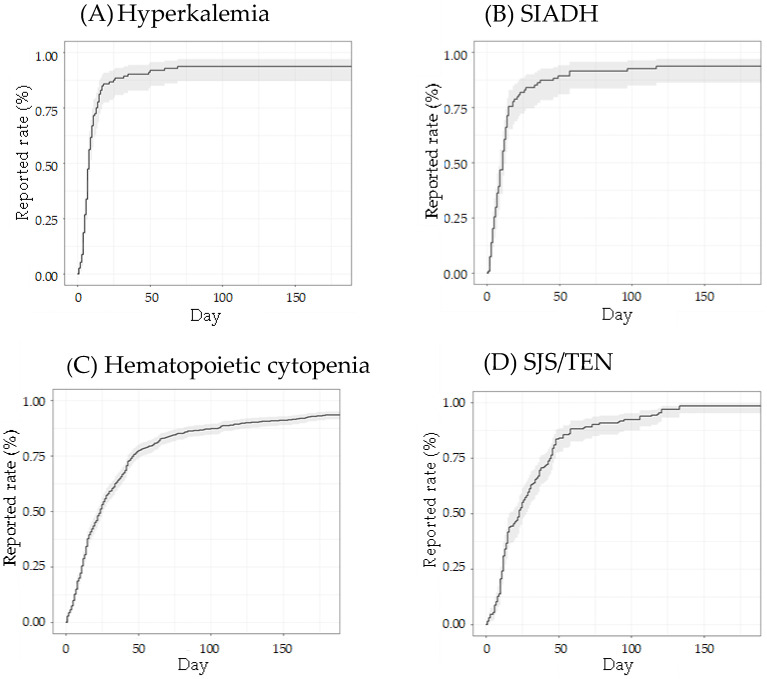

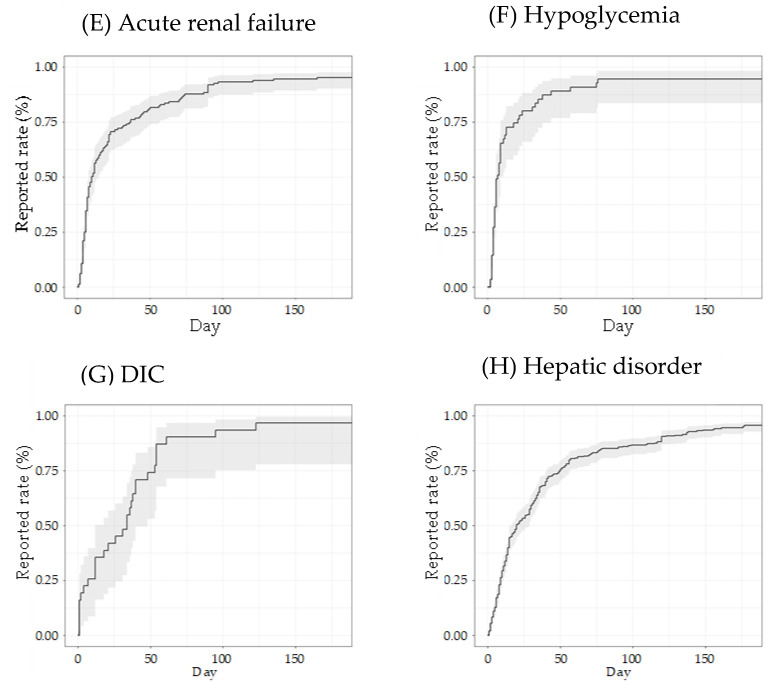
Cumulative incidence of adverse events over time. Kaplan–Meier-like curves illustrate the time to reporting of (**A**) hyperkalemia, (**B**) syndrome of inappropriate secretion of antidiuretic hormone, (**C**) hematopoietic cytopenia, (**D**) Stevens–Johnson syndrome/toxic epidermal necrolysis, (**E**) acute renal failure, (**F**) hypoglycemia, (**G**) disseminated intravascular coagulation, and (**H**) hepatic disorder following drug administration, based on data from the spontaneous reporting system. The x-axis represents the number of days from the start of drug administration to adverse event onset, and the y-axis indicates the cumulative reported rate. Shaded areas represent 95% confidence intervals.

The analysis of the time from the start of ST administration to the onset of adverse events is shown in Figure 1. The change in cumulative incidence over time for ST combination agents is illustrated in the Kaplan–Meier plot (Figure 2). The median (IQR) onset times (days) for ST-related adverse events were as follows: hyperkalemia (8 (5–13)), SIADH (11 (5–15)), hematopoietic cytopenia (24 (11–45)), SJS/TEN (23 (12–44)), acute renal failure (11 (6–40)), hypoglycemia (8 (4–23)), and hepatic disorder (21 (9–52)). All adverse events occurred within the first 2 months of treatment. Time-to-onset analysis of adverse events by ST administration is shown in Figure 1.

Weibull shape parameters for ST-related adverse events were as follows: hyperkalemia (α = 16, 95% CI: 11–21; β = 0.66, 95% CI: 0.59–0.73), SIADH (α = 28, 95% CI: 18–43; β = 0.50, 95% CI: 0.44–0.57), hematopoietic cytopenia (α = 46, 95% CI: 41–51; β = 0.71, 95% CI: 0.68–0.75), SJS/TEN (α = 38, 95% CI: 31–46; β = 0.76, 95% CI: 0.70–0.83), acute renal failure (α = 33, 95% CI: 25–45; β = 0.59, 95% CI: 0.53–0.65), hypoglycemia (α = 28, 95% CI: 16–47; β = 0.52, 95% CI: 0.43–0.61), and hepatic disorder (α = 45, 95% CI: 39–53; β = 0.72, 95% CI: 0.67–0.77). All events were categorized as early failures, with an upper limit of 95% CI and β values < 1, indicating a lower failure rate over time.

## 4. Discussion

The JADER database has been increasingly utilized to investigate rare and serious adverse drug reactions that are challenging to evaluate through single-center or prospective cohort studies. For instance, previous studies have used JADER to assess the risk of pseudoaldosteronism associated with the traditional Japanese medicine yokukansan, as well as central nervous system ischemia linked to bevacizumab use [21,26]. These studies highlight the utility of JADER in detecting safety signals for adverse events that may be underrepresented in clinical trials or routine observational studies. Building on this approach, the present study is the first to use the JADER to systematically evaluate adverse events associated with ST, with a particular focus on their frequency and onset timing. Signals were detected for several serious adverse events, including SJS/TEN, SIADH, and hypoglycemia. In a logistic regression model adjusted for age, gender, and year of report as confounders for each adverse event with ST as the suspected drug, the adverse events for which signals were detected in adjusted ROR were consistent with those identified in the crude ROR. This consistency suggests that the observed signals are unlikely to be substantially confounded by age, gender, or reporting year. An evaluation of the onset timing of adverse events using the Weibull distribution suggested that these events occur within the first 2 months of drug administration.

Through the inhibition of folate synthesis, trimethoprim has been linked to dose-dependent bone marrow suppression [3,4,5]. In this study, adverse event signals related to myelosuppression were also identified through analysis of the JADER database. The median time of occurrence of myelosuppression-related events was 24 days. Since folate deficiency occurs within 1 month of folate synthesis inhibition [27], the onset date of folate deficiency should coincide with that of thrombocytopenia. ST has also been associated with renal failure due to the inhibition of tubular transporters and hepatic disorders caused by immunogenicity. In this study, analysis of the JADER database similarly detected signals for hematopoietic cytopenia, renal failure, and hepatic disorders.

ST has been reported to cause side effects such as renal dysfunction, SIADH, and hyperkalemia [14,15]. However, trimethoprim is known to inhibit creatinine excretion by blocking the organic cation transporter expressed in the proximal tubules, and there are reports that creatinine blood levels are only transiently increased, independent of kidney function [28]. Therefore, it has remained unclear whether ST causes renal dysfunction. In this study, we investigated these adverse events and detected signals for renal dysfunction, SIADH, and hyperkalemia associated with ST use. Overall, 317 cases of renal dysfunction were reported with ST as the suspect drug. It is known that transient elevations in creatinine are unlikely to be reported in cases where actual renal function is unaffected [29]. Additionally, there have been reports that ST metabolites, owing to their low solubility, can precipitate in urine and cause hematuria, interstitial nephritis, and tubular necrosis [30,31,32]. Based on the above, it is possible that ST affects renal function through mechanisms that are not yet fully understood. The effect of ST on renal dysfunction and its underlying mechanism should be further investigated in future studies. In this study, the time of renal dysfunction onset was 11 (6–41) days. In previous reports, interstitial nephritis has been shown to develop within approximately 1 week of initiation of dosing, which is consistent with our findings [5,32]. It has also been reported that trimethoprim induces hyponatremia and hyperkalemia by inhibiting Na+/K+-ATPase in the distal tubule [33]. Furthermore, trimethoprim is structurally similar to amiloride and is thought to act on epithelial sodium channels in the distal nephron, leading to natriuresis and hyponatremia [15]. Therefore, the hyperkalemia caused by ST may not be secondary to impaired renal function but rather an electrolyte abnormality mediated by transporter inhibition. In the present study, hyperkalemia and SIADH occurred at 11 (5–15) days, most often shortly after taking the drug. Considering that these electrolyte abnormalities are due to transporter inhibition, we believe this onset time is reasonable [15].

Additionally, although case reports of ST-induced liver injury exist [34], the actual situation remains unclear because no large-scale investigative study has been conducted. Drug-induced liver injury can be broadly classified into predictable and unpredictable (idiosyncratic) types [34]. In the case of ST-induced liver injury, it has been suggested that idiosyncratic mechanisms, possibly related to genetic polymorphisms, may be involved [35]. Previous studies have reported that patients with ST-induced liver injury more commonly carry HLA gene polymorphisms, particularly HLA-B*14:01 and HLA-B*35:01, which are known to readily bind sulfamethoxazole [35]. This suggests that sulfamethoxazole forms immune complexes with HLA, activating T cells and leading to hepatocellular damage. Hepatocellular-type liver injury is known to require an incubation period ranging from several days to up to 90 days from the start of use of the suspect drug [34]. The time of onset of hepatocellular damage caused by the ST combination drug in this study was 21 days (9–52 days), which aligns with the presumed mechanism of action.

A case report suggested that ST use may induce severe adverse events, such as anaphylaxis and SJS/TEN, through immunogenic mechanisms [36]. However, few studies have been conducted, and measures to address this issue remain unclear.

Drug package inserts in various countries include warnings; however, the lack of sufficient research has led to differing views.

In Japan, the drug package insert includes warnings about anaphylactic reactions, while in the United States and the United Kingdom, SJS/TEN is listed as a serious adverse event [18,19,20]. In this study, SJS/TEN was detected as a suspected adverse drug reaction, whereas no signal was detected for anaphylactic reactions. These findings suggest that SJS/TEN is more likely to occur as an immunogenic adverse event induced by ST use. Furthermore, similar immunogenic mechanisms have also been reported with other antibiotic classes, such as β-lactams, macrolides, and fluoroquinolones, which have been implicated in severe cutaneous adverse reactions, including SJS/TEN. However, sulfonamides, particularly sulfamethoxazole, have shown a stronger association with these reactions, likely caused by the formation of highly reactive metabolites and specific HLA risk alleles such as HLA-B13:01 and HLA-A11:01, which are more prevalent in certain populations [8].

The proposed mechanism through which SJS/TEN occurs involves the binding of sulfamethoxazole nitroso metabolites to T cells, inducing an immune response based on previous in vitro findings [37]. Drug eruptions caused by type IV hypersensitivity reactions have a delayed onset, with symptoms appearing from one week to several months after the initiation of treatment once sensitization occurs [38]. The time of onset of SJS/TEN in this study was 23 days (IQR: 12–45 days), which is consistent with the hypothesis that SJS/TEN is caused by type IV hypersensitivity.

In addition, basic research using rats has reported that trimethoprim induces hyponatremia and hyperkalemia by inhibiting sodium-potassium ATPase activity in the distal tubules [33]. Trimethoprim, structurally similar to amiloride, has been suggested to affect epithelial sodium channels in the distal nephron, leading to natriuresis and hyponatremia [15]. In this study, signals for SIADH and hyperkalemia were also detected, suggesting that these adverse events can occur in clinical practice. The onset time for these adverse events was 11 days (IQR: 5–15 days) for both hyperkalemia and SIADH, with most cases occurring within the first week after administration. Given that these electrolyte abnormalities are caused by transporter inhibition, the onset time is considered reasonable [15].

The time-to-onset analysis in the 2359 patients with available data showed that serious adverse events, such as hyperkalemia, SIADH, and hypoglycemia, occurred mainly within 2 weeks after treatment initiation, with symptoms occurring as early as 5 days. These findings suggest that electrolyte monitoring, particularly of potassium and sodium levels, should begin within 7 days of therapy, especially in patients with renal dysfunction, older individuals, and those taking concomitant medications that affect electrolyte balance. Furthermore, since these serious events tend to occur early in the course of treatment, it is recommended that ST be prescribed with caution in outpatient settings, with prompt follow-up after the first week and regular monitoring of laboratory values to assess the patient’s condition.

Several case reports have linked the use of ST to severe and potentially fatal hypoglycemia [17,39]. However, no large-scale studies have been conducted, and the clinical characteristics remain unclear [17]. In this study, a signal for hypoglycemia was detected. Previous reports suggest that the sulfonamide structure of sulfamethoxazole induces adenosine triphosphate-sensitive potassium channels and insulin secretion in mice, which may explain the clinical hypoglycemia observed [40]. Sulfonamide derivatives typically cause hypoglycemia within a few hours of administration [41]; therefore, it is likely that ST-induced hypoglycemia can also occur rapidly. The results of this study showed an onset time of 8 days (4–23 days), which is consistent with the expected mechanism of action.

The causative factors of adverse events were classified into dose-dependent and immune-mediated categories. Hematopoietic cytopenia, SIADH, hyperkalemia, and hypoglycemia were more frequently observed in cases with higher daily or cumulative doses, suggesting a dose-dependent mechanism. In contrast, SJS/TEN and hepatic disorders tended to occur regardless of the administered dose, indicating an immune-mediated mechanism.

ST-related adverse events exhibit both regional consistency and variability across global data sources. Hematologic toxicities are consistently reported as common in cohort and pharmacovigilance studies conducted in Asia, Europe, and North America [42,43,44]. However, SIADH and hypoglycemia, which generated strong signals in our JADER analysis, have been rarely reported in Western literature, suggesting potential under-recognition outside Japan. Conversely, SJS/TEN, though rare, is a well-documented serious event across all regions [45,46]. Notably, anaphylaxis is reported more frequently in Japanese case series and reports but is less commonly emphasized in reports from Europe and North America. Pharmacogenomic studies have further identified HLA alleles, such as HLA-B13:01, HLA-A11:01, and HLA-B*15:02, as significant risk factors for sulfonamide-induced severe cutaneous adverse reactions in East Asian populations [47]. This genetic predisposition may partly account for the heightened incidence and regulatory emphasis on severe cutaneous reactions observed in East Asia. These findings underscore the importance of globally harmonized safety information and suggest that SIADH, hypoglycemia, and anaphylaxis may warrant closer epidemiological and pharmacovigilance assessment outside Japan.

### Limitations

This study had certain limitations. First, although we used a spontaneous adverse event reporting database, owing to the nature of the database, information on pathogens prompting ST use, comorbidities, and clinical laboratory data was unavailable. The absence of these potential confounding factors may have affected the interpretation of our findings.

Second, the JADER database is subject to inherent limitations associated with spontaneous reporting systems, including underreporting, duplicate submissions, and selection bias [48,49]. Duplicate reports referring to the same case were excluded in this study; however, underreporting remains a significant limitation, as not all suspected adverse events are reported to the PMDA. Moreover, the database includes only reports from patients who experienced adverse events, which may result in a study population that does not fully represent the general clinical population. Additionally, this study did not involve a control group, limiting its ability to draw causal inferences through comparative analysis. These factors may introduce bias in signal detection and restrict the generalizability of the findings. Therefore, our results should be interpreted with caution and further validated through prospective studies that include case and control groups, such as cohort studies and randomized controlled trials.

Third, a high percentage of serious adverse drug reactions tend to be reported in JADER, including a high number of anaphylaxis reports [48]. In this study, 39,117 anaphylaxis reports were recorded in the entire JADER dataset, accounting for 4.5% of all reports. Therefore, the high number of anaphylaxis reports associated with drugs other than ST, coupled with the relatively low number of reports linked to ST, may explain why no signal was detected for ST. This suggests that anaphylaxis reports are relatively infrequent among ST-related adverse events but does not rule out the possibility that anaphylaxis can occur with ST. Therefore, it is necessary to investigate the frequency of anaphylaxis in actual clinical practice and compare its occurrence with that of comparable antimicrobial agents in future studies.

In this study, the identification of side effects specific to ST was enabled, as well as the timing of adverse events. Among them, no signal was detected for anaphylactic reactions, which is warned against in the Japanese package insert. On the other hand, hematopoietic cytopenia, for which a similar warning exists, was detected. As hematopoietic cytopenia is a dose-dependent side effect, it has recently been suggested that the incidence of hematopoietic cytopenia can be suppressed by adjusting the dosage of the drug. However, the JADER database contains many missing data points on dosage, making it challenging to investigate this possibility fully. Therefore, it is necessary to clarify the relationship between dosage and the occurrence of adverse effects through prospective studies.

Consequently, further validation through cohort studies and randomized controlled trials is required. Nevertheless, our study, by utilizing a spontaneous adverse event reporting database, contributes valuable insights into the frequency and timing of adverse events associated with ST.

## 5. Conclusions

In this study, adverse events such as hematopoietic cytopenia, acute renal failure, hypoglycemia, and SIADH were observed to occur shortly after ST administration. These findings emphasize the need for early monitoring and risk management during treatment. They also highlight the need to revise the Japanese ST package insert to include warnings for hypoglycemia and SIADH. Future prospective studies and international pharmacovigilance efforts incorporating genetic risk factors, such as HLA alleles, are warranted to validate these associations and support the global harmonization of safety information.

## Figures and Tables

**Table 1:** Signal detection of each adverse event with sulfamethoxazole/trimethoprim.

Adverse Event	Suspected					Concomitant + Interaction				
	Case	No Case	Ratio (%)	ROR (95%CI)	*p* Value	Case	No Case	Ratio (%)	ROR (95% CI)	*p* Value
Hyperkalemia	230	3973	5.5	11.3 (9.85–13.0)	<0.001	110	19,073	0.6	1.08 (0.88–1.31)	0.42
SIADH	136	4067	3.2	4.67 (3.90–5.55)	<0.001	188	18,995	1.0	1.37 (1.18–1.58)	<0.001
Hematopoietic cytopenia	1232	2971	29.3	3.53 (3.31–3.79)	<0.001	5744	13,439	29.9	3.79 (3.67–3.91)	<0.001
Cytopenia affecting more	311	3892	7.4	4.22 (3.75–4.75)	<0.001	909	18,274	4.7	2.68 (2.50–2.88)	<0.001
Erythropenia	43	4160	1.0	4.06 (2.93–5.50)	<0.001	102	19,081	0.5	2.12 (1.72–2.59)	<0.001
Leukopenia	701	3502	16.7	2.80 (2.58–3.04)	<0.001	4147	15,036	21.6	4.05 (3.91–4.20)	<0.001
Thrombocytopenia	334	3869	7.9	2.56 (2.28–2.86)	<0.001	2267	16,916	11.8	4.20 (4.01–4.39)	<0.001
Hypersensitivity	1167	3036	27.8	2.78 (2.59–2.97)	<0.001	1651	17,532	8.6	0.67 (0.64–0.71)	<0.001
SJS/TEN	343	3860	8.2	7.19 (6.41–8.04)	<0.001	149	19,080	0.8	0.61 (0.51–0.72)	<0.001
Anaphylactic reaction	46	4157	1.1	0.23 (0.17–0.31)	<0.001	522	18,661	2.7	0.58 (0.53–0.64)	<0.001
Acute renal failure	317	3886	7.5	2.39 (2.12–2.68)	<0.001	713	18,470	3.7	1.13 (1.04–1.21)	0.002
Hypoglycemia	100	4103	2.4	2.33 (1.89–2.84)	<0.001	84	18,089	0.5	0.41 (0.33–0.51)	<0.001
DIC	47	4156	1.1	2.25 (1.64–2.99)	<0.001	188	18,995	1.0	1.99 (1.71–2.31)	<0.001
Hepatic disorder	589	3614	14.0	2.07 (1.90–2.26)	<0.001	1487	17,696	7.8	1.07 (1.01–1.12)	0.021
Interstitial lung disease	165	4038	3.9	0.88 (0.75–1.03)	0.12	855	18,328	4.5	1.01 (0.94–1.08)	0.79
Nausea and vomiting symptoms	55	4148	1.3	0.81 (0.61–1.06)	0.14	327	18,856	1.7	1.06 (0.95–1.19)	0.28
Noninfectious diarrhea	45	4158	1.1	0.78 (0.57–1.05)	0.11	380	18,803	2.0	1.47 (1.33–1.63)	<0.001
Rhabdomyolysis	26	4177	0.6	0.69 (0.45–1.01)	0.059	58	19,125	0.3	0.33 (0.25–0.43)	<0.001

Abbreviations: 95% CI, 95% confidence interval; DIC, disseminated intravascular coagulation; ROR, reporting odds ratio; SIADH, syndrome of inappropriate secretion of antidiuretic hormone; SJS/TEN, Stevens–Johnson syndrome/toxic epidermal necrolysis.

**Table 2:** Adjusted reporting odds ratio of each adverse event with sulfamethoxazole/trimethoprim.

Adverse Events	Adjusted ROR (95% CI)	*p*-Value
Hyperkalemia	9.45 (8.10—1.10)	<0.001
SIADH	4.75 (3.98—5.65)	<0.001
Hematopoietic cytopenia	3.56 (3.32—3.81)	<0.001
Cytopenia affecting more	4.35 (3.87—4.90)	<0.001
Erythropenia	3.65 (2.65—5.02)	<0.001
Leukopenia	2.79 (2.57—3.03)	<0.001
Thrombocytopenia	2.45 (2.19—2.76)	<0.001
Hypersensitivity	2.66 (2.48—2.86)	<0.001
SJS/TEN	6.74 (6.01—7.56)	<0.001
Anaphylactic reaction	0.20 (0.15—0.28)	<0.001
Acute renal failure	2.21 (1.95—2.50)	<0.001
Hypoglycemia	2.41 (1.96—2.96)	<0.001
Hepatic disorder	1.98 (1.81—2.17)	<0.001
Interstitial lung disease	0.88 (0.75—1.03)	0.11
Nausea and vomiting symptoms	0.79 (0.60—1.04)	0.092
Noninfectious diarrhea	0.78 (0.58—1.05)	0.099
Rhabdomyolysis	0.58 (0.39—0.88)	0.010

Abbreviations: 95% CI, 95% confidence interval; ROR, reporting odds ratio; SIADH, syndrome of inappropriate secretion of antidiuretic hormone; SJS/TEN, Stevens–Johnson syndrome/toxic epidermal necrolysis.

## Data Availability

The datasets generated and analyzed during the current study are available from the corresponding author upon reasonable request.

## Abbreviations

The following abbreviations are used in this manuscript:

CI	Confidence interval
IDSA	Infectious Diseases Society of America
IQR	Interquartile range
JADER	Japanese Adverse Drug Event Report
MedDRA	Medical Dictionary for Regulatory Activities
PMDA	Pharmaceuticals and Medical Devices Agency
PT	Preferred term
ROR	Reported odds ratio
SIADH	Syndrome of inappropriate antidiuretic hormone secretion
SJS/TEN	Stevens–Johnson syndrome/toxic epidermal necrolysis
ST	Sulfamethoxazole/trimethoprim

## Appendix A

**Table A1:** Top reported adverse effects of sulfamethoxazole/trimethoprim.

Preferred Term Code	Case (*n*)
10019670 Hepatic function abnormal	237
10035528 Platelet count decreased	237
10020646 Hyperkalaemia	211
10033661 Pancytopenia	210
10037844 Rash	199
10001507 Agranulocytosis	198
10042033 Stevens–Johnson syndrome	198
10073508 Drug reaction with eosinophilia and systemic symptoms	187
10013687 Drug eruption	187
10047942 White blood cell count decreased	174
10062237 Renal impairment	168
10022611 Interstitial lung disease	165
10044223 Toxic epidermal necrolysis	141
10024670 Liver disorder	131
10015218 Erythema multiforme	125
10069339 Acute kidney injury	120
10029354 Neutropenia	112
10021036 Hyponatraemia	110
10029366 Neutrophil count decreased	108
10043554 Thrombocytopenia	100
10016288 Febrile neutropenia	89
10020993 Hypoglycaemia	84
10072268 Drug-induced liver injury	77
10002034 Anaemia	71
10038428 Renal disorder	62
10015150 Erythema	61
10028584 Myelosuppression	52
10024384 Leukopenia	47
10013442 Disseminated intravascular coagulation	47
10012735 Diarrhoea	45
10025256 Lymphocyte count decreased	43
10028813 Nausea	40
10003481 Aspartate aminotransferase increased	35
10001551 Alanine aminotransferase increased	32
10066274 Cytopenia	30
10047700 Vomiting	28
10048302 Tubulointerstitial nephritis	27
10039020 Rhabdomyolysis	26
10071583 Haemophagocytic lymphohistiocytosis	26
10060795 Hepatic enzyme increased	26

**Table A2:** Annual counts of total adverse event reports and reports related to sulfamethoxazole/trimethoprim.

Reporting Year	Total Case (*n*)	Suspected ST, *n* (%)	Concomitant + Interaction ST, *n* (%)
2004	24,403	118 (0.48)	379 (1.55)
2005	24,168	103 (0.43)	454 (1.88)
2006	24,281	110 (0.45)	568 (2.34)
2007	25,610	157 (0.61)	543 (2.12)
2008	28,593	149 (0.52)	571 (2.00)
2009	29,297	165 (0.56)	843 (2.88)
2010	32,972	169 (0.51)	1167 (3.54)
2011	36,173	175 (0.48)	1364 (3.77)
2012	40,778	194 (0.48)	999 (2.45)
2013	37,927	221 (0.58)	1026 (2.71)
2014	48,451	231 (0.48)	904 (1.87)
2015	50,191	241 (0.48)	1282 (2.55)
2016	54,929	272 (0.50)	1174 (2.14)
2017	60,263	365 (0.61)	1303 (2.16)
2018	61,472	320 (0.52)	1219 (1.98)
2019	60,604	298 (0.49)	1108 (1.83)
2020	51,843	253 (0.49)	1100 (2.12)
2021	82,353	318 (0.39)	1374 (1.67)
2022	71,877	283 (0.39)	1530 (2.13)
2023 (first quarter)	16,767	61 (0.36)	275 (1.64)
Total (2004–2023)	862,952	4203 (0.49)	19,183 (2.22)

Abbreviation: ST, sulfamethoxazole/trimethoprim.
